# Characterization of Growth and Cell Cycle Events Affected by Light Intensity in the Green Alga *Parachlorella kessleri*: A New Model for Cell Cycle Research

**DOI:** 10.3390/biom11060891

**Published:** 2021-06-15

**Authors:** Vilém Zachleder, Ivan N. Ivanov, Veronika Kselíková, Vitali Bialevich, Milada Vítová, Shuhei Ota, Tsuyoshi Takeshita, Shigeyuki Kawano, Kateřina Bišová

**Affiliations:** 1 Laboratory of Cell Cycles of Algae, Centre Algatech, Institute of Microbiology of the Czech Academy of Sciences, 37981 Třeboň, Czech Republic; zachleder@alga.cz (V.Z.); ivanov@alga.cz (I.N.I.); kselikova@alga.cz (V.K.); bialevich@alga.cz (V.B.); vitova@alga.cz (M.V.); 2 Faculty of Science, University of South Bohemia, 37005 České Budějovice, Czech Republic; 3 Center for Environmental Biology and Ecosystem Studies, National Institute for Environmental Studies, Tsukuba, Ibaraki 305-8506, Japan; ota.shuhei@nies.go.jp; 4 The University of Tokyo Future Center Initiative, Wakashiba 178 4 4, Kashiwa, Chiba 277-0871, Japan; takeshita@algalbio.co.jp (T.T.); kawano@edu.k.u-tokyo.ac.jp (S.K.)

**Keywords:** cell cycle pattern, energy reserves, growth processes, light intensity, *Parachlorella kessleri*, reproduction events, deuterium, deuterated starch, deuterated lipid

## Abstract

Multiple fission is a cell cycle variation leading to the production of more than two daughter cells. Here, we used synchronized cultures of the chlorococcal green alga *Parachlorella kessleri* to study its growth and pattern of cell division under varying light intensities. The time courses of DNA replication, nuclear and cellular division, cell size, total RNA, protein content, dry matter and accumulation of starch were observed at incident light intensities of 110, 250 and 500 µmol photons m^−2^s^−1^. Furthermore, we studied the effect of deuterated water on *Parachlorella kessleri* growth and division, to mimic the effect of stress. We describe a novel multiple fission cell cycle pattern characterized by multiple rounds of DNA replication leading to cell polyploidization. Once completed, multiple nuclear divisions were performed with each of them, immediately followed by protoplast fission, terminated by the formation of daughter cells. The multiple fission cell cycle was represented by several consecutive doublings of growth parameters, each leading to the start of a reproductive sequence. The number of growth doublings increased with increasing light intensity and led to division into more daughter cells. This study establishes the baseline for cell cycle research at the molecular level as well as for potential biotechnological applications, particularly directed synthesis of (deuterated) starch and/or neutral lipids as carbon and energy reserves.

## 1. Introduction

Growth of algae is affected by a few main factors: (1) temperature, that affects the kinetics of the entire metabolism, (2) the source of energy and (3) the source of carbon. For autotrophically grown algae in nature, the levels of carbon dioxide, their carbon source, are stable. Algae compensate for a lack of sufficient carbon dioxide in the air by exploiting carbon concentration mechanisms [1,2]. Nevertheless, increasing levels of CO_2_ will improve growth rates and thus can be used in algal biotechnology. Light and temperature are the two main factors that fluctuate the most and which affect growth rates of light-grown algae. Light serves as the main energy source; with increasing light intensity, growth rates will increase [3,4] until the light reaches inhibitory levels, whereupon growth is negatively affected. In chlorococcal and volvocean algae, including *Parachlorella kessleri*, increasing light intensity will also induce the formation of larger mother cells and lead to the production of more daughter cells [3,4]. The production of more than two daughter cells is typical for their cell cycle pattern, multiple fission. Such cell cycles consist of several growth steps [5,6,7,8,9] that are separated in time and do not overlap. Their length is affected by growth rate under any given cultivation condition (intensity of light and temperature, no growth occurs in dark). At the end of each growth step, the cells attain a commitment to trigger a sequence of reproductive events, which, in due time, are terminated even in the absence of energy supply, i.e., in the dark [3,4]. The multiple fission cell cycle is characterized by several (n) rounds of reproductive sequence each consisting of DNA replication, nuclear and cellular divisions, which is completed by formation of 2^*n*^ daughter cells (for details, see review [3]). The number of daughter cells formed from a single mother cell (division number) depends on light and temperature. It will increase with increase in light intensity as well as with prolongation of light period [3,4], yet it has certain limits. The cell cycle is composed of a growth period of variable length depending on the growth conditions and a reproductive period with a length set by the duration of one or several rounds of reproductive sequences. The two are coordinated and in optimal conditions usually lead to division into 8 or 16 cells. There are at least two distinct types of multiple fission, a consecutive pattern (Scenedesmus—type cell cycle) and a clustered pattern (Chlamydomonas—type cell cycle) [3,6]. These two multiple fission cell cycle patterns share stepwise growth characteristics but differ in timing of individual processes within the reproductive sequences. DNA replication and nuclear divisions for individual reproductive sequences either happen consecutively, one by one (Scenedesmus type) or happen for all the reproductive sequences in a very short time frame at the end of the cell cycle (Chlamydomonas type). Thus, the main morphological difference between the two is the presence (Scenedesmus type) or absence (Chlamydomonas type) of multinuclear stages within the cell cycle.

The chlorococcal alga *Parachlorella kessleri* (formerly *Chlorella kessleri* [10]), has, within a relatively short time period, become frequently used as a model experimental organism for diverse research activities and biotechnological applications [11,12,13,14,15,16,17,18,19,20,21,22,23]. It has particularly attracted interest because, depending on growth conditions, it can produce both starch and neutral lipid [16,24]. Both of these energy reserves can be (over)produced, which makes this alga an interesting organism for algal biotechnology. The primary energy store for the alga is starch. It is produced under optimal growth conditions in complete nutrient medium, at physiological values of light intensities and temperatures [16,25]. Moreover, carbon partitioning is affected by stress conditions leading to starch accumulation. Starch can (over)accumulate under nutrient limitation such as nitrogen or sulfur depletion [18,19,26], high light intensity [27] or in the presence of a high concentration of CO_2_ [28]. Neutral lipid, the secondary energy reserve, under optimal growth conditions, can be kept relatively low at 1–10% of dry matter (DM) [16,24,25]. It can also (over)accumulate under stress conditions similar to these inducing starch accumulation such as nitrogen, sulfur or phosphorus depletion [18,20,24,26,29], dilution of all the nutrients in medium [19,24] or salt stress [30]. The neutral lipids tend to accumulate later than the peak of starch accumulation [18,24,26]. One of the stress conditions known to induce neutral lipid (over)accumulation is growth in deuterated water [31]. Deuterium, the stable isotope of hydrogen is known to have the highest kinetic effect among the stable isotopes of biogenic elements [31,32,33]. It affects the entire metabolism and its specific effects range from disrupting signaling [34] and energy producing production in mitochondria and chloroplasts [35,36] to disrupting cell division [37,38]. The treatment with deuterated water is quite expensive and artificial as such high concentrations of deuterated water are never present in nature. Yet, it can be justified for production of fine (bio)chemicals with very high added value. Algae-derived deuterated biomolecules such as carotenoids, lipids, and starch, are of interest both as analytical standards and for metabolic labeling [31,39,40].

Although extensive knowledge has accumulated on starch and neutral lipid production under different conditions, basic information on *P. kessleri* growth under non-limiting growth conditions is missing. It is unclear how growth is affected by external growth conditions such as irradiance and temperature and how its cell cycle is organized. This obviously hinders further exploitation of this alga for biotechnology. The current work provides baseline information on the effect of different light intensities on growth (RNA, protein, dry matter, cell volume), the reproductive steps of the cell cycle (DNA replication, nuclear division, protoplast fission, formation and liberation of daughter cells), and accumulation of energy and carbon reserves (starch). We aimed to establish the division pattern of *Parachlorella kessleri* as well as the effect of light on progression of the cell cycle. Furthermore, we tested the effect of deuterated water as a specific stressor on both growth and cell cycle progression in *P. kessleri*. Such information can serve as a starting point for further optimization of the alga for biotechnology purposes as well as its exploitation as a novel model in basic research.

## 2. Materials and Methods

### 2.1. Organism and Culture

The green unicellular microalga *Parachlorella kessleri* (Trebouxiophyceae, Chlorophyta), (strain CCALA 255), was obtained from the Culture Collection of Autotrophic Organisms at the Institute of Botany, Czech Academy of Sciences in Třeboň, Czech Republic (CCALA; http://www.butbn.cas.cz/ccala/index.php, accessed on 7 June 2021).

For routine sub-culturing, the cultures were streaked every three weeks onto nutrient medium (see below) solidified by agar (1.5%) and grown on a light shelf at an incident light intensity of 100 μmol photons m^−2^s^−1^ of photosynthetically active radiation.

**Figure 1:**
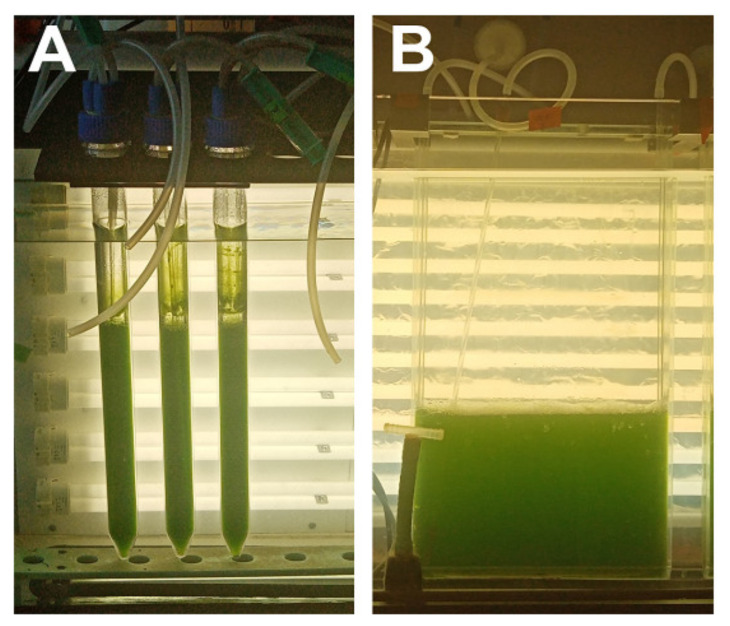
Laboratory photo-bioreactors used for the experiments. (**A**) glass cylinders (300 mL), (**B**) plane-parallel cuvettes (2500 mL).

For experiments, laboratory cultivation units consisted of glass cylinders (inner diameter 36 mm, height 500 mm, volume of suspension 300 mL), or glass vessels, which were flat and rectangular in shape (inner dimensions 400 × 300 × 20 mm^3^, volume of suspension 2500 mL) (Figure 1). Culture units were placed in a thermostatic water bath (30 ± 0.5 °C) and continuously illuminated by a panel of dimmable fluorescent lamps (DULUX L55W/950 Daylight, OSRAM, Munich, Germany) with maximum incident light intensity of 750 μmol photons m^−2^s^−1^. They were vigorously mixed with air bubbles containing 2% carbon dioxide (*v/v*) dispersed through a 200 µL micropipette tip fixed to the glass tube placed at the bottom of the cylinder. The aeration in flat vessels was done by bubble stream from a perforated stainless steel tube located at the bottom of the vessel. The flow-rate of the aeration mix was 60 L/h.

### 2.2. Mineral Nutrient Medium

The mineral medium was based on the mean content of P, N, K, Mg, and S in algal biomass [41] and had the following initial composition (in mg/L): 2020 KNO_3_, 237 KH_2_PO_4_, 204 MgSO_4_·7H_2_O, 40 C_10_H_12_O_8_N_2_NaFe, 88 CaCl_2_, 0.83 H_3_BO_3_, 0.95 CuSO_4_·5H_2_O, 3.3 MnCl_2_·4H_2_O, 0.17 (NH_4_)_6_Mo_7_O_24_·4H_2_O, 2.7 ZnSO_4_·7H_2_O, 0.6 CoSO_4_·7H_2_O, and 0.014 NH_4_VO_3_ in distilled water [27]. For preparation of medium, 100x concentrated stock solutions of macro-elements and microelements were used. All components were diluted in distilled water and autoclaved for 30 min at 121 °C. The pH was adjusted to 7 with 1 M NaOH.

### 2.3. Synchronization of Cultures

The cultures were grown under optimal conditions: incident light intensity 500 μmol photons m^−2^s^−1^, temperature 30 °C, 2% CO_2_ in aeration mixture. To set up the synchronization regime, the culture was grown in continuous light until the cell concentration reached approximately 1 × 10^6^ cells mL^−1^. Next, the culture was put into dark to allow division of all the cells that were capable to do so. Once the cells within the culture were divided, the cultures were put on light and carefully observed by light microscopy for two or three cycles to set the correct length of both the light and dark periods under given conditions. The lengths of light and dark periods were chosen according to the growth parameters of the cells. The time for darkening the cells was when about 10% of cells started their first protoplast fission. The length of the dark period was chosen to allow all cells of the population to release their daughter cells and then the durations of the light and dark periods were kept constant. With the number of light/dark cycles, the synchrony of the cells increased until it was possible to set up the light/dark regime to be used for synchronization. During the synchronization procedure, the cell density was kept below 1 × 10^6^ cells mL^−1^ by dilution at the end of dark period to prevent cell shading. By this treatment, the synchronization was set to 18 h of light followed by 7 h of dark. This light/dark regime was used throughout the experiments such that the cells were inoculated into the growth media and grown at this light/dark regime until the cell density reached 1 × 10^6^ cells mL^−1^ whereupon it was kept at this cell density by dilution at the end of dark period until the volume of the culture required for the start of the experiment was reached. The synchronized daughter cells were again diluted to the initial cell density approximately 1 × 10^6^ cells mL^−1^ and used as inocula for experimental cultures.

### 2.4. Measurement of Light Intensity

A quantum/radiometer-photometer (LI-COR, Inc., Lincoln, NE, USA) was used. In the culture unit, dimmable fluorescent tubes were used for adjustment of irradiance. To obtain a measure of light energy absorbed by a layer of cell suspension grown at different incident light intensities (I_i_) and different optical densities (concentrations of cells), the mean light intensity (I_m_) was calculated according to the Lambert-Beer formula: I_m_ = (I_i_ − I_t_)/ln(I_i_/I_t_), where I_i_ is the incident light intensity measured at the surface of the culture vessel, and I_t_ is the transmitted light intensity measured at the rear side of the culture vessel.

### 2.5. Assessment of Nuclear and Cell Division Curves

The proportion of mother cells, and daughter cells was determined by light microscopy in cells fixed in Lugol solution (1 g I, 5 g KI, 100 mL H_2_O) at a final concentration 10 μL of Lugol solution per 1 mL of cell suspension.

### 2.6. Dry Matter Determination

Biomass was separated from the medium by sequential centrifugation of 4 mL of the cell suspension in pre-weighed microtubes at 3000× *g* for 5 min; the sediment was dried at 105 °C for 12 h and weighed on an analytical balance (TE214S-0CE, Sartorius, Goettin-gen, Germany) [27].

### 2.7. Cell Volume and Number

Cell volume and concentration were measured using a Beckman Coulter Multisizer 4 (Beckman Coulter Life Sciences, Brea, CA, USA) by diluting 50 µL of fixed (0.2% glutaraldehyde) cell suspension into 10 mL of 0.9% NaCl (*w/v*) electrolyte solution.

### 2.8. Nuclei and Cell Wall Staining

Nuclei were stained with SYBR Green and observed through a fluorescent microscope using the method described by Vítová, et al. [42], for details see also Hlavová, et al. [43]. Five microliters of freshly defrosted cell pellet were combined with 2.5 µL of SYBR Green I (cat. no. S7563, ThermoFisher, Waltham, MA, USA) and 2.5 µL of Fluorescent Brightener 28 (cat. no. 910090, equivalent to Calcofluor, Sigma-Aldrich, St. Luis, MO, USA), vortexed and kept for 5–10 min in the dark at room temperature. Next, the cells were observed using a LSM Zeiss 880 confocal microscope (Carl Zeiss, Jena, Germay) using the following excitation wavelength: chlorophyll—633 nm, Fluorescent Brightener 28—488 nm and SYBR Green I—405 nm.

### 2.9. Quantum Yield Measurement

Aliquots of 2 mL were withdrawn from the culture and placed into 10 × 10 mm^2^ plastic cuvettes for 30 min in the dark. Quantum yield was measured using an AquaPen-C AP-C 100 (Photon Systems Instruments, Drasov, Czech Republic).

### 2.10. Measurement of Neutral Lipid Content

Neutral lipid content was measured spectrophotometrically in a microplate format following the modified procedure of Takeshita, et al. [44]. Aliquots (100 μL) of the cultures were transferred to a 96-well plate and 5 μL of freshly prepared Nile Red dye (0.5 mg/mL in DMSO, catalog no. 72485, Sigma-Aldrich, Prague, Czech Republic) were added to each well. The same amount of Nile Red dye was added to a sample blank consisting of 100 μL of H_2_O. The plate was incubated at room temperature for 15 min. Fluorescence was measured using an Infinite 200 PRO microplate reader (Tecan, Männedorf, Switzerland) equipped with a 485 nm excitation filter and a 595 nm emission filter. Fluorescence intensity of the samples was normalized using fluorescence intensity of unstained samples and a blank. Standard curves produced from a commercial lipid standard, triolein (catalog no. Y0001113, Sigma-Aldrich, St. Luis, MO, USA) were used to quantify neutral lipids.

### 2.11. Estimation of Bulk RNA, DNA and Protein

#### 2.11.1. Total Nucleic Acids

The procedure of Wanka [45] as modified by Lukavský, et al. [46] was used for the extraction of the total nucleic acids. The samples were centrifuged in 10 mL centrifuge tubes, which also served for storage of the samples. The pellet of algal cells was stored under one ml of ethanol at −20 °C. The pellet was extracted 5 times with 0.2 M perchloric acid in 50% ethanol for 50 min at 20 °C and 3 times with an ethanol-ether mixture (3:1) at 70 °C for 10 min. Such pre-extracted samples can be stored in ethanol at −20 °C. Total nucleic acids were extracted and hydrolyzed by 0.5 M perchloric acid at 60 °C for 5 h. After hydrolysis, concentrated perchloric acid was added to achieve a final concentration of 1 M perchloric acid in the sample. Absorbance of total nucleic acids in the supernatant was read at 260 nm (A_260_) and total nucleic acid concentration was calculated based on the calibration with DNA standard of the known concentration treated by the same procedure.

#### 2.11.2. DNA and RNA Determination

The light activated reaction of diphenylamine with hydrolyzed DNA, as described by Decallonne and Weyns [47] was used with the following modification of Zachleder [48]: the diphenylamine reagent (4% diphenylamine in glacial acetic acid, *w/v*) was mixed with the samples of the total nucleic acid extracts in a ratio of 1:1 and the mixture in the test tubes was illuminated from two sides with fluorescent lamps (L 40W F33, Tungsram, Budapest, Hungary). The incident radiation from each side was 150 μmol photons m^−2^s^−1^. After 6 h of illumination at 40 °C, the difference between the A_600_ and A_700_ nm was estimated. The concentrations of DNA within the samples were set by comparison to the A_600_ and A_700_ nm values of the sample with known DNA concentration treated the same way. The values were normalized to the number of cells extracted. The RNA content was calculated as the difference between the total nucleic acid and DNA content.

#### 2.11.3. Protein Determination

The sediment remaining after nucleic acid extraction was used for protein determination. It was hydrolyzed by 1 M NaOH for 1 h at 70 °C. The protein concentration in the supernatant after centrifugation of the hydrolysate (15 min, 5300× *g*, room temperature) was estimated by BCA assay (cat. no. 23225, Thermo Fisher Scientific, Waltham, MA, USA) according to manufacturer’s conditions. The same procedure was carried out with a calibration curve set by different concentrations of bovine serum albumin.

### 2.12. Starch Analyses

Cell pellets containing approximately 2 × 10^6^ cells mL^−1^ were harvested during the cell cycle, washed with SCE buffer (100 mM sodium citrate, 2.7 mM EDTA-Na_2_, pH 7 (citric acid)), snap frozen in liquid nitrogen and stored at −20 °C. After thawing the cells, the pellets were disintegrated by adding 300 µL of zirconium beads (0.7 mm in diameter) and vigorously vortexing for 15 min (Vortex-Genie 2, Scientific Industries Inc., Bohemia, NY, USA). Depigmentation of the samples was done by adding 1 mL of 80% (*v/v*) ethanol to the pellet and incubating in a water bath (SW22, Julabo, Seelbach, Germany) for 15 min at 68 °C, after which the samples were centrifuged for 2 min at 14,000 × *g* and the supernatant was removed. The depigmentation procedure was repeated 3 to 4 times (or until the pellet was completely discolored).

After that 1 mL of α-amylase from porcine pancreas (Sigma-Aldrich, St. Luis, MO, USA), (0.5 mgL^−1^ (*w/v*) in 0.1 M sodium phosphate buffer (pH 6.9)) was added to the samples and they were incubated for 1 h at 37 °C (FTC 90i, VELP Scientifica, Usmate Velate, MB, Italy). The samples were centrifuged for 2 min at 14,000× *g*, after which the supernatant was used for the quantification of reducing sugars through the DNSA color reaction as described in [49]. In short, 500 µL of supernatant were mixed with 500 µL dinitrosalicylic acid (DNSA) solution (1% (*w/v*) 3,5- DNSA, 30% (*w/v*) potassium sodium tartrate tetrahydrate, 20% (*v/v*) 2M sodium hydroxide) and incubated for 5 min at 105 °C on a heat block. Following a cooling period of 10 min at room temperature the mixture was diluted five-fold with distilled water, after which the A_570_ of the samples were measured. The concentration of starch was estimated through a calibration curve of potato starch (Lach-Ner, Neratovice, Czech Republic) digested with α-amylase.

### 2.13. Statistical Analysis

Experiments were performed in at least three biological replicates. If not stated otherwise, all results are presented as means and standard deviation (*n* = 3).

## 3. Results

### 3.1. Light Characteristics

**Figure 2:**
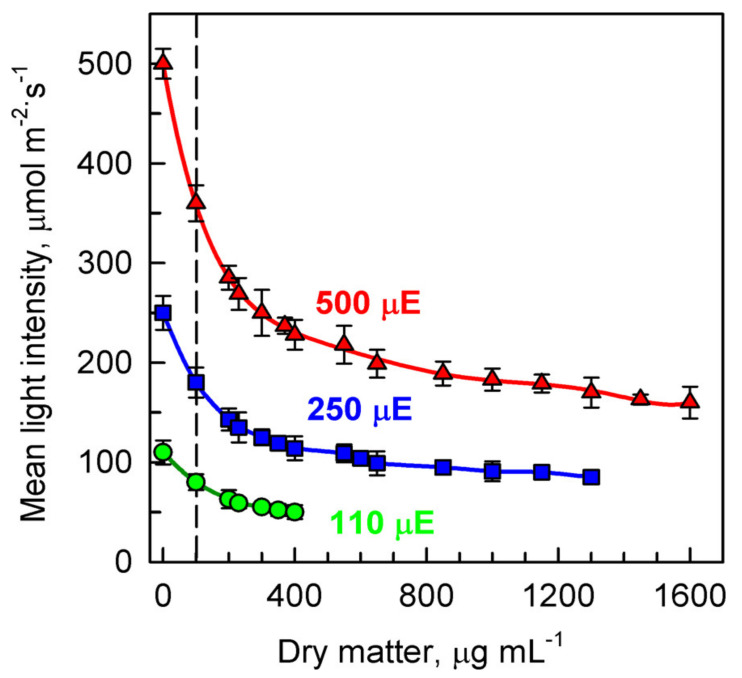
Changes in mean light intensity with increasing dry matter during the cell cycle of synchronous cultures of *Parachlorella kessleri* grown at different incident light intensities of 500, 250, 110 μmol photons m^−2^s^−1^ (μE) at a temperature of 30 °C. Culture growth was started at the same initial dry matter (100 µg mL^−1^), indicated by the vertical dashed line.

Light intensity and cell growth are interconnected so that increasing cell size and cell numbers will decrease the light availability to the cells and hence affect growth. To establish the relationship between light intensity and dry matter yield, cultures were illuminated at the following incident light intensities: 500, 250, 110 μmol photons m^−2^s^−1^ (Figure 2). However, the incident light intensity only represents the light at the surface of the bioreactor, which is absorbed by the first layer of cells in the suspension. Instead, mean light intensity provides a biologically meaningful value of light conditions in the cultures. It is calculated from incident and transmitted light intensities (see Methods) and reflects the effect of dry matter density. The dry matter density is a function of cell concentration and cell size. To avoid the effects of cell density, the same initial biomass density (dry matter, cell size and cell number) was applied at the beginning of the experiments (0 h of the cell cycles) at a given incident light intensity. The initial concentration of dry matter at the beginning of the cell cycle was 100 µg mL^−1^ (Figure 2, horizontal axis, vertical dashed line). The mean light intensities, relative to the incident light intensities of 500, 250, 110 μmol photons m^−2^s^−1^, were 360, 180, and 80 μmol photons m^−2^s^−1^ respectively. With ongoing culture growth, dry matter increased until the final values attained at the end of the cell cycles were 1600, 1300 and 400 µg mL^−1^ respectively. This caused an exponential decrease in the mean light intensity so that at the end of cell cycles it had dropped to 160, 85, 50 μmol photons m^−2^s^−1^ respectively (Figure 2).

### 3.2. Time Course of Cell Cycle Characteristics

**Figure 3:**
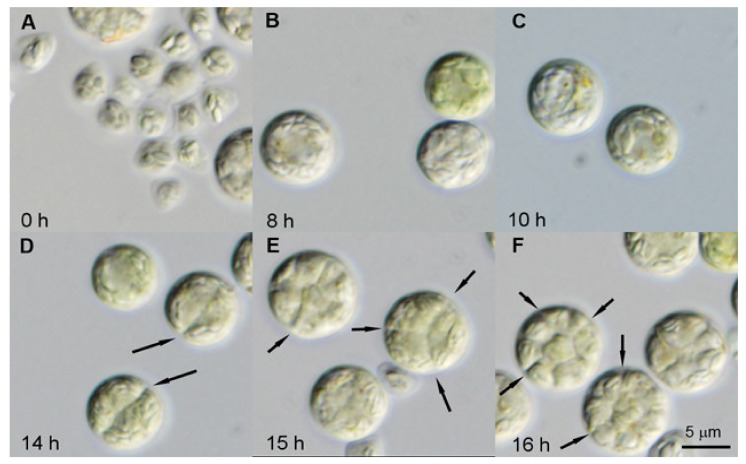
Course of cell cycle progression in synchronized cells of *Parachlorella kessleri* grown at a light intensity of 250 μmol photons m^−2^s^−1^ and a temperature of 30 °C. Light photo-micrographs of daughter cells at 0 (**A**), 8 h (**B**) and 10 h (**C**) and mother cell dividing their protoplast into 2 ((**D**)—14 h), 4 ((**E**)—15 h), and into 8 ((**F**)—16 h). Division furrows are marked by arrows. Scale = 5 µm.

**Figure 4:**
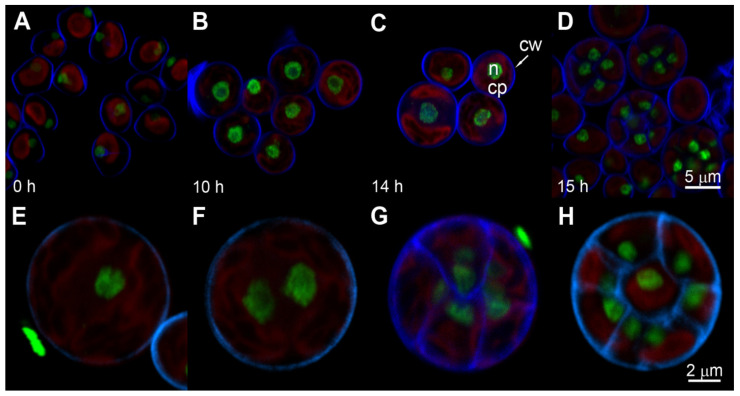
Fluorescent photo-micrographs of synchronized cells of *Parachlorella kessleri* grown in light at 250 μmol photons m^−2^s^−1^ and a temperature of 30 °C at 0 h (**A**), 10 h (**B**)**,** 14 h (**C**) and 15 h (**D**) of the cell cycle. Magnified cells show dividing nuclei, chloroplasts and protoplasts into 2 (**E**,**F**), 8 (**G**) and 16 (**H**) daughter cells. To visualize nuclei, the cells were stained by fluorescent dye SYBR Green I dye, the cell walls were stained by calcofluor (Fluorescent Brightener 28) dye. Green stained nucleus, n; red autofluorescence of chloroplast, cp; blue stained cell wall, cw and arrow. Scale is 5 µm for (**A**–**D**), and 2 µm for (**E**–**H**).

Daughter cell cultures used as the initial inocula were obtained by synchronization under optimal growth conditions (see Material and Methods). The morphological changes during the cell cycle are shown on the example of synchronized daughter cells grown at the incident light irradiance of 250 μmol photons m^−2^s^−1^ (Figure 3). Daughter cell cultures consisted of small cells (mean cell volume 25 µm^3^) (Figure 3A, 0 h) with a single tiny nucleus (Figure 4A, 0 h). The cultures at the beginning of the cell cycle sometimes contained both small triangular shaped released daughter cells and divided daughter cells still present in division clusters (Figure 3A). It is common that some of the daughter cells stay connected in the division clusters in dark and immediately after being put to light, but they split into individual daughter cells within 1–2 h of cultivation in light. Other cultures were formed exclusively by individual split cells from the beginning of the experiment (Figure 4A). Neither of these conditions affect the progression through the cell cycle. The cell size increased from the beginning of the cell cycle (Figure 3A, 0 h) to about 14 h when the first protoplast fission into two daughter cells started (Figure 3D, 14 h). Thereafter, within two hours, protoplast fission into four (Figure 3E, 15 h) and then into final eight daughters occurred (Figure 3F, 16 h). The daughter cells formed their own cell walls inside the mother cells (Figure 4D). The mother cell wall was then broken and the daughter cells were liberated. The cells transferred into dark during protoplast fission formed and liberated the daughter cells as in the light but were not growing any more.

The nuclear staining showed there was no nuclear division until about 14 h but nuclei increased in size (Figure 4A–C). An increasing size of nuclei indicated an increase in nuclear DNA content (for more details see below). The enlarged, polyploid nuclei underwent multiple consecutive divisions at the very end of the cell cycle. Each of the mitoses was concurrently or immediately followed by chloroplast and protoplast fissions (compare Figure 3D–F and Figure 4C–H).

### 3.3. Effect of Light Intensities

During cultivation at different light intensities the course of reproductive events was followed, i.e., DNA replication, nuclear division, protoplast fission and daughter cell formation at different light intensities. This was supplemented by assessing different growth parameters such as accumulation of bulk RNA and protein and increased cell size, as well as changes in the level of starch as the primary energy and carbon reserve.

#### 3.3.1. Sequence of Reproductive Events

**Figure 5:**
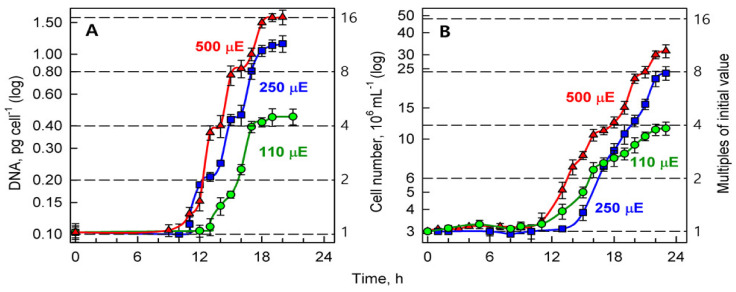
The course of reproductive events in synchronized cultures of *Parachlorella kessleri* at the optimal temperature of 30 °C and different incident light intensities (500, 250, 110 μmol photons m^−2^s^−1^) (red triangles, blue squares, green circles respectively). Panel (**A**): DNA (pg cell^−1^), Panel (**B**): cell number (10^6^ mL^−1^). Horizontal dashed lines indicate doublings of the initial values at the beginning of the cell cycle.

The synchronized culture at the beginning of the cell cycle consisted of a single small nucleus (Figure 4A) containing 0.1 pg of DNA (Figure 5A). The sequence of several DNA-duplications started after about 10 h of growth. Individual duplications of DNA (DNA replication rounds) were separated by about a 2-h period without any DNA synthesis and the number of duplications per cell increased with light irradiance applied (Figure 5). At the highest incident light intensity (500 μmol photons m^−2^s^−1^) four rounds of DNA replication occurred, leading to a 16-fold increase in the DNA content of the daughter cell (from 0.1 pg cell^−1^ to 1.6 pg cell^−1^) (Figure 5A, triangles). At a medium incident light intensity (250 μmol photons m^−2^s^−1^), a 16-fold increase was attained only by about half of the cell population and only three doublings of DNA were performed in the second half of population, as shown by a 12-fold increase in DNA content (Figure 5A, squares). Slow growth at the lowest incident light intensity used (110 μmol photons m^−2^s^−1^) enabled only 2 doublings of DNA content and the cells contained only 4-fold higher DNA content compared to their initial values. With decreasing light intensity, the start of consecutive DNA replication rounds was delayed for 2 h at 250 and 4 h at 110 μmol photons m^−2^s^−1^ when compared to DNA replication in the highest replication light intensity cultures (Figure 5A). Because nuclear division did not follow immediately after DNA, the size of nuclei increased proportionally to the increasing content of DNA, becoming consecutively di-, tetra-, octu- or hexadecaploid but the cells remained uni-nuclear (Figure 4 and Figure 5).

Shortly after all DNA replications were completed, the number of nuclear divisions occurred, corresponding to the preceding number of rounds of DNA replication. This was concomitant with chloroplast division and was immediately followed by protoplast fissions, as only bi-nuclear but no poly-nuclear cells were observed (Figure 4D–H). After completing multiple protoplast fissions, daughter cells were formed and released (Figure 4D and Figure 5B).

In general, light intensity was, at a given temperature, a decisive factor affecting the number of rounds of DNA replication (doublings of DNA content), which were performed during the cell cycle. The number of DNA replication rounds then determined the number of nuclear divisions, the number of protoplast fissions and the number of daughter cells released, corresponding to 2^*n*^, where *n* is the number of doublings. In *P. kessleri,* at a given temperature, *n* varies from 1 to 4, and so from 2 to 16 daughter cells could be released at the end of the cell cycle. The number *n* increased with light intensity. In the experimental conditions tested, the lowest number of daughter cells was 4 but a lower light intensity would be required for the formation of fewer daughter cells.

#### 3.3.2. Sequence of Growth Event

**Figure 6:**
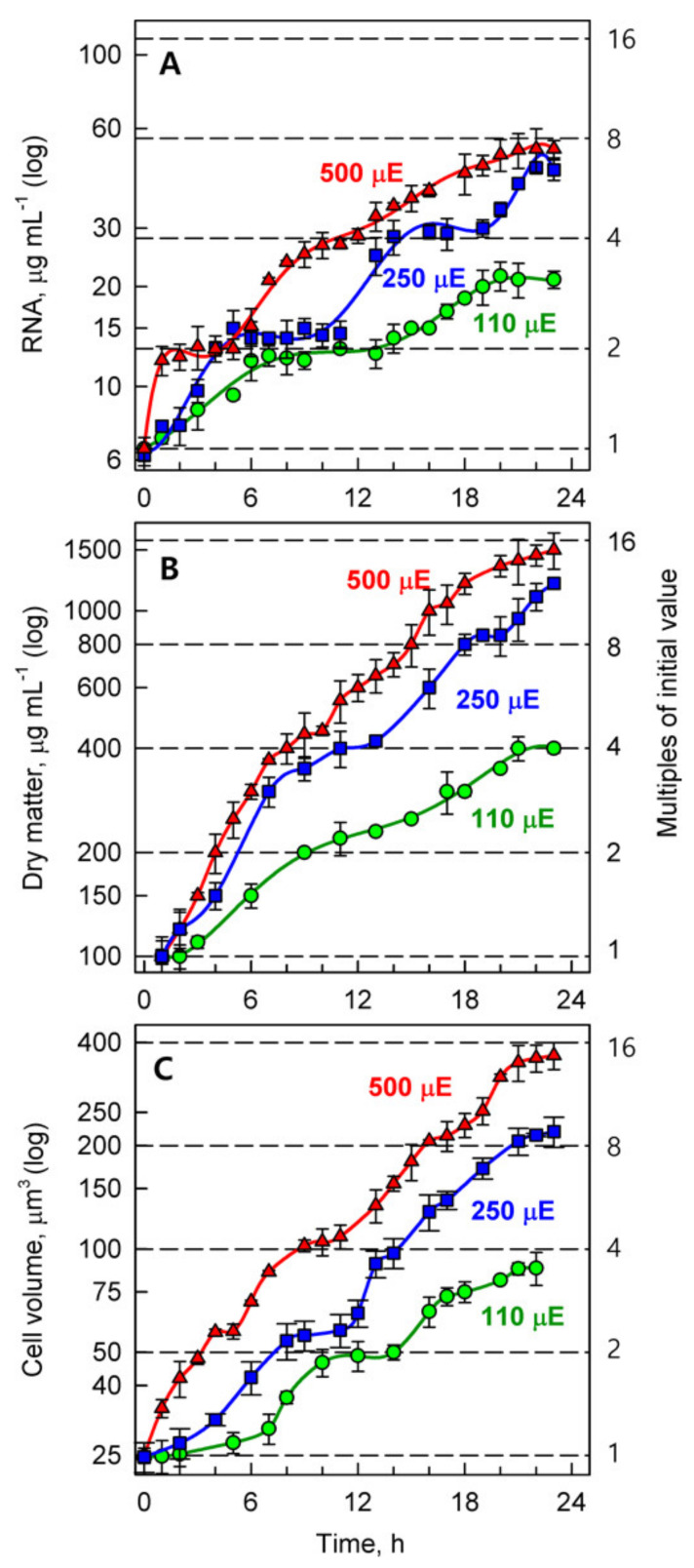
Time course of growth events in the synchronized cultures of *Parachlorella kessleri* grown at the optimal temperature of 30 °C and different incident light intensities (500 (red triangles), 250 (blue squares), 110 (green circles) μmol photons m^−2^s^−1^). Panel (**A**): RNA (μg mL^−1^), Panel (**B**): Dry matter (μg mL^−1^), Panel (**C**) Cell volume (μm^3^). Note the logarithmic scale on the Y axis. Horizontal dashed lines indicate doublings of the initial values.

To assess the growth of the cultures, three growth parameters were monitored: bulk RNA content, dry matter and cell volume. All of the growth parameters were the highest at the highest light intensity of 500 μmol photons m^−2^s^−1^ (Figure 6) and decreased both in values and rates with decreasing light intensity, from 250 to 110 μmol photons m^−2^s^−1^ (Figure 6). The growth events increased in several steps, each of which corresponded to approximately a doubling of the initial/preceding values (indicated by horizontal dashed lines in Figure 6), and led to a 2- to 16-fold increase in the initial values (Figure 6). The growth steps were separated by intervals without growth (4–6 h), as is particularly well documented for an increase in RNA content (Figure 6A). With increasing growth rate, the intervals without growth shortened and, in fast growing cells, sometimes became undistinguishable (Figure 6B, see the first doubling at 500 and 250 μmol photons m^−2^s^−1^).

Only two doublings (4-fold increase) occurred in all growth characteristics at the lowest light intensity (110 μmol photons m^−2^s^−1^) (Figure 6). With increasing light intensity, three doublings of growth parameters were attained at 250 μmol photons m^−2^s^−1^. At a light intensity of 500 μmol photons m^−2^s^−1^, there occurred four doublings in cell volume and dry matter (Figure 6B,C) but only three doublings of RNA content (Figure 6A).

#### 3.3.3. Energy Reserves

**Figure 7:**
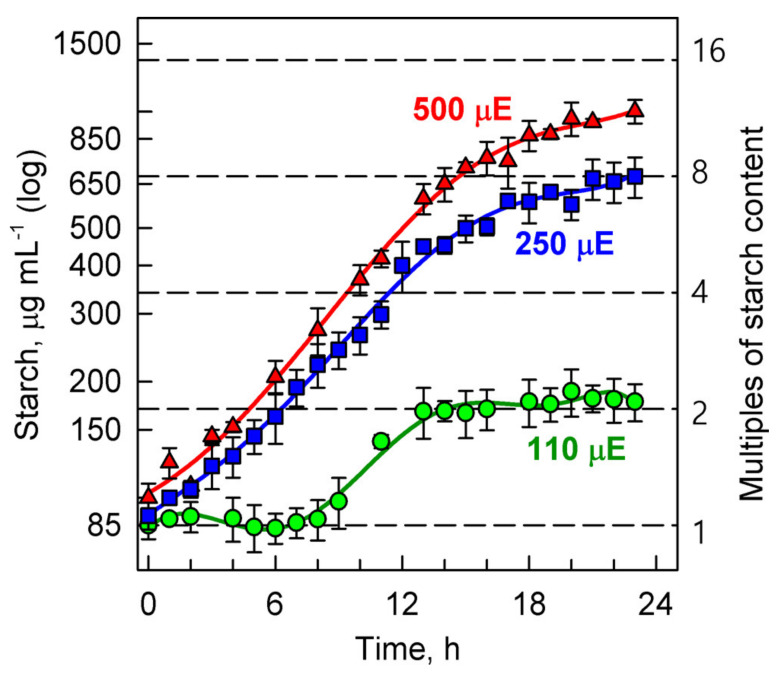
Time course of starch accumulation in synchronized cultures of *Parachlorella kessleri* at the optimal temperature of 30 °C and different incident light intensities (500, 250, 110 μmol photons m^−2^ s^⁠^^−1^) (red triangles, blue squares, green circles respectively). Horizontal dashed lines indicate doublings of starch content compared with the beginning of the cell cycle.

The synthesis of starch was affected similarly to cell growth parameters; its content increased with increasing light intensity. At low incident light intensity (110 μmol photons m^−2^s^−1^) the net content of starch only doubled, starting to increase not earlier than in the second half of the cell cycle (6 h), and after cell division (12 h), its level did not increase further; the starch content remained constant (Figure 7).

At higher light intensity, the net starch accumulation started from the very beginning of the cell cycle and continued with increasing rate until cell division (12 h). Then it slowed but did not stop. At the end of the cell cycle, the cells contained 8-fold higher levels of starch at the incident light intensity of 250 μmol photons m^−2^s^−1^ and about 10-fold higher at 500 μmol photons m^−2^s^−1^ compared to the initial values (Figure 7).

### 3.4. Cell Division and Growth at Different Light Intensities in Deuterated Water

**Figure 8:**
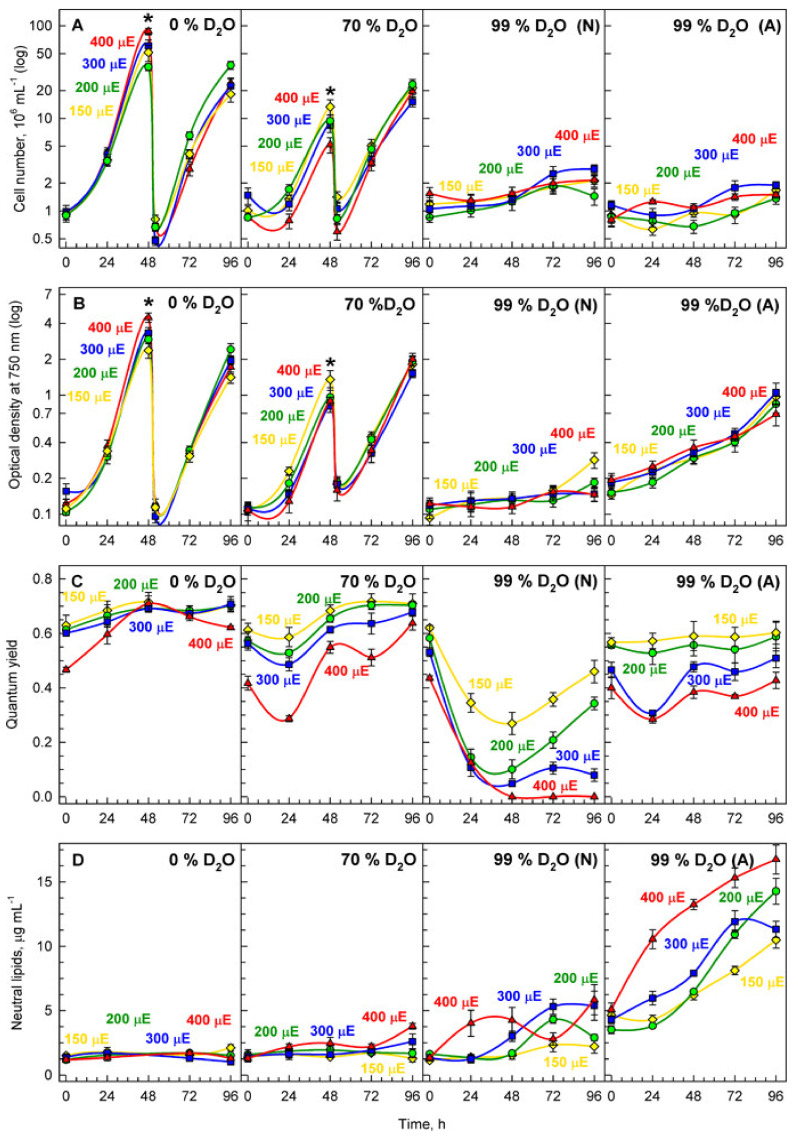
Time course of cell division and growth in synchronized cultures of *Parachlorella kessleri* grown at the optimal temperature of 30 °C, different incident light intensities (400 (red triangles), 300 (blue squares), 200 (green circles), and 150 (yellow diamonds) μmol photons m^−2^s^−1^) and different concentrations of deuterated water in the medium (0, 70 and 99%). The cultures transferred directly from control conditions into 99% deuterated water are denoted as “N”, the cultures pre-adapted at the same concentration for one week are denoted as “A”. Panel (**A**): cell number (10^6^ mL^−1^), Panel (**B**): Optical density at 750 nm, Panel (**C**): quantum yield, Panel (**D**): neutral lipids (µg mL^−1^). Note the logarithmic scale on Y axis for A and B.

As shown in the previous experiments, increasing light intensity caused a rise in both growth rates and the number of daughter cells, suggesting a distinct beneficial effect. Deuterated water has been known to negatively affect growth of all organisms [31,32] by increasing the level of cellular stress. To study its effect on *P. kessleri*, nutrient medium containing increasing concentrations of deuterated water (0, 70, and 99%) was used; this was combined with different incident light intensities (150, 200, 300 and 400 μmol photons m^−2^s^−1^). We analyzed how their combination affected cell division, growth, and the physiological state of the culture (Figure 8).

#### 3.4.1. Cell Division

In the control culture without any deuterated water in the medium, the cells divided approximately every 24 h and the average number of newly formed daughter cells (division number) increased with increasing incident light intensity from less than 6 to more than 9 daughter cells formed (Figure 8A, Table 1). The cell division number was not affected by initial cell concentration up to approximately 4 × 10^6^ cells mL^−1^. This corresponds approximately to the cell density of 3 × 10^6^ cells mL^−1^ used in the experiments above (Figure 2, Figure 3, Figure 4, Figure 5, Figure 6 and Figure 7). To avoid the decrease in mean light intensity due to cell shading, the cultures were periodically diluted to a concentration of 1 × 10^6^ cells mL^−1^ once they reached optical density at 750 nm greater than 1. By this sequential dilution approach, we were able to follow four consecutive cell cycles in the control cultures, about three consecutive cell cycles in 70% cultures and about one cell cycle for the 99% cultures (Figure 8A). In the individual cell cycles, there was negligible difference in number of formed daughter cells as is clear from the comparison among the number of daughter cells formed in the first 24 h (and again between 48 and 72 h) and between 24 and 48 h (and again between 72 and 96 h). This also suggested that continuous light did not significantly affect the division patterns. In the presence of 70% *v/v* deuterated water in the medium (70% culture), the division number decreased to approximately 4 under all light intensities (Figure 8A, Table 1). This represented a decrease compared to the control culture in all variants. The decrease was more pronounced with increasing light intensities (Figure 8A, Table 1). Cultivation in 99% *v/v* deuterated water (99% culture) further decreased the division number to below 2 (Figure 8A, Table 1). Similarly to the 70% culture, reduction in the division number was more pronounced with increasing light intensities. To determine whether adaptation of cells to high concentrations of deuterated water could improve the cells reaction, the cultures were pre-adapted to 99% *v/v* deuterated water in the medium prior to the experiment. Such pre-adapted cultures showed similar reductions in division number as the non-adapted culture but the doubling times were shorter compared to the non-adapted culture (Table 1). However, the culture was prone to high death rates at higher light intensities, probably due to extensive oxidative stress.

#### 3.4.2. Cell Growth

Due to the inhibitively high price of deuterated water, cell growth was assessed as changes in optical density at 750 nm, i.e., outside of the main chlorophyll absorbance peaks and corresponding mostly to light scattering (Figure 8B). In the control culture, biomass multiplied more with increasing light intensity and increasing growth rate although it multiplied less than cell number (Figure 8A,B, compare Table 1 and Table 2). The mass doubling time shortened with increasing light intensity but the correlation between the two was less pronounced than for cell doubling time. In the 70% culture, mass multiplication was reduced compared to the controls but the reduction was lower than that of the division number (Figure 8A,B, compare Table 1 and Table 2). The mass doubling times were comparable for all light intensities and with the exception of the highest light intensity, were higher than the cell number doubling times (Figure 8, compare Table 1 and Table 2). In the 99% culture, mass multiplication factor was significantly affected compared to the control cultures and the reduction was clearer with increasing light intensity (Figure 8, Table 2). With the exception of the lowest light intensity, mass multiplication factor was comparable to the corresponding changes in division number (compare Table 1 and Table 2). The mass doubling time was reduced to about 10% (light intensity 150 and 200 μmol photons m^−2^s^−1^) or about 5% (light intensity 300 and 400 μmol photons m^−2^s^−1^) (Table 2). The reduction was thus similar to the reduction in cell division doubling time (compare Table 1 and Table 2). In the pre-adapted culture, the reduction in mass multiplication factor followed similar trends as that in the non-adapted culture but the reduction was lower (Figure 8B, Table 2). Furthermore, the reduction was lower than that of division number (compare Table 1 and Table 2).

#### 3.4.3. Stress

From above, it is clear that deuterium causes stress leading to both a decline in cell division and a slowing down of growth. Quantum yield, in dark-adapted samples equivalent to the ratio between maximum fluorescence and variable fluorescence, Fv/Fm, was used to assess the stress levels in individual cultures over time (Figure 8C). In the control culture, the cells experienced stress immediately after being put in light but with time, recovered from it (Figure 8C). In the 70% culture, there was a significant degree of variation in Fv/Fm over time. This was most expressed at the highest light intensity (400 μmol photons m^−2^s^−1^) (Figure 8C). Firstly, the ratio dropped to very low level as stress was experienced by all cultures. Next, the ratio in all the cultures started to recover suggesting the cells adapted to the stress caused by the treatment. The non-adapted cells grown in the medium containing 99% deuterated water experienced severe stress at all light intensities although it was lower at the lowest light intensity (150 μmol photons m^−2^s^−1^) (Figure 8C). Recovery from stress was light intensity dependent and it started only after five days of cultivation (Figure 8). In line with this, the cells pre-adapted to 99% deuterated water showed stress compared to the control culture but for the two lower light intensities (150 and 200 μmol photons m^−2^s^−1^) it remained similar throughout the experiment (Figure 8). For the two higher light intensities (300 and 400 μmol photons m^−2^s^−1^), the cultures experienced higher levels of stress but recovered within 48 h (Figure 8C).

#### 3.4.4. Starch and Neutral Lipid Accumulation

**Figure 9:**
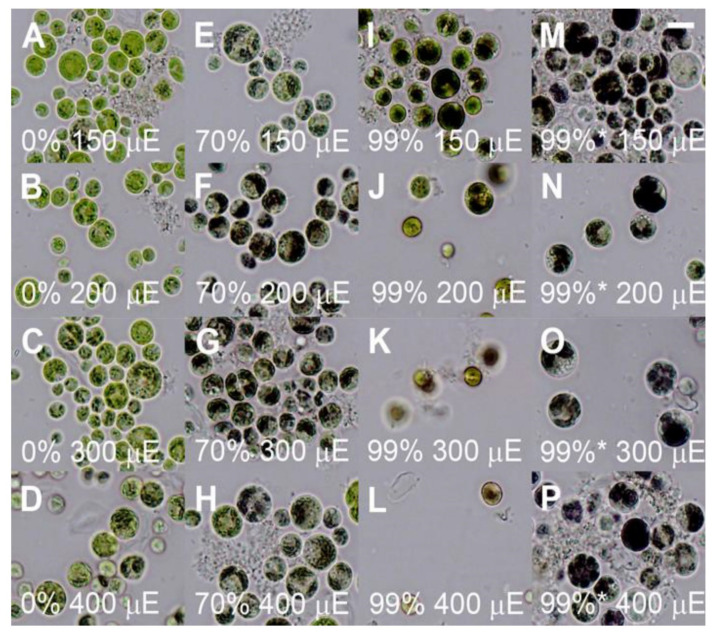
Light photo-micrographs of *Parachlorella kessleri* grown for 3 days at different incident light intensities (400, 300, 200, and 150 μmol photons m^−2^s^−1^) and different concentrations of deuterated water in the medium (0, 70 and 99%). (**A**–**D**): control cultures grown in 0% D_2_O, (**E**–**H**) cultures grown in 70% D_2_O, (**I**–**L**) cultures grown in 99% D_2_O, (**M**–**P**) cultures grown in 99% D_2_O and pre-adapted to the same concentration for one week. The cultures pre-adapted to growth at 99% of deuterated water for one week are denoted with asterisk. Scale is 10 µm.

Cultures of *P. kessleri* are known to produce varying amounts of starch and neutral lipids in response to stress [16,26,31,50]. We tested whether the combination of increasing light intensity and the presence of increasing concentrations of deuterated water in the medium could induce starch and/or neutral lipid accumulation. The neutral lipid accumulation was negligible in the control cultures grown in 0% D_2_O as well as in the 70% cultures (Figure 8D). But it was increased in both the non-adapted and pre-adapted 99% cultures (Figure 8D). In the pre-adapted cultures, there was a higher accumulation of neutral lipids with increasing light intensity (Figure 8D). Due to the inhibitive price of deuterated water, starch accumulation was assessed on cells stained by Lugol solution (Figure 9). In the control cultures, there was more starch produced with increasing light intensities (Figure 9A–D) as is also shown above (Figure 7). In the 70% culture, the accumulation of starch was higher than in the controls and comparable among all the light intensities (Figure 9E–H). In the 99% culture, the highest accumulation of starch was reached at the lowest light intensity (Figure 9I). It was lower at higher light intensities (Figure 9J–L) concomitant with higher neutral lipid accumulation in these cells (Figure 8D). From all the variants, the accumulation of starch was highest in the pre-adapted culture (Figure 9M–P) and it was comparable across the light intensities.

## 4. Discussion

*Parachlorella kessleri* is a biotechnologically relevant algal species that, depending on the growth conditions, is able to (over)produce both starch and neutral lipids [16,26]. A cell cycle division block has been shown to be one of the mechanisms leading to both starch and neutral lipid production [16,26,50]. However, the pattern of cell division under optimal non-stressed growth conditions remained unknown. Here, we characterized the cell cycle pattern and studied the effect of light intensity on cell cycle progression. To do so, we used synchronized cultures where all the cells within the population were in approximately the same phase of the cell cycle. This is evidenced by stepped increases in DNA content in the entire population and the sharp increases in cell number (Figure 5A,B). Furthermore, the cell volume is giving an idea how the cell size increased within the population with time (Figure 6C). For non-synchronous cultures, it would be expected instead that the lines describing the changes in cell numbers and DNA content will be parallel with abscissa as both processes would be running continuously in the cell population. Moreover, we characterized the effect of different light intensities on growth, including the levels of energy reserves. Finally, we tested how cell division and growth were affected by the presence of deuterium. The synchronization by light/dark periods used here is a natural process. The algae dividing by multiple fission evolved so that they exploit the alternating light/dark periods of solar irradiation to grow during the light phase and divide during the dark phase [3,4]. Still, once all the reproductive processes were completed, the cells would divide irrespective if they were in dark or on light (Figure 5B and Figure 8A) [51,52]. Once the culture is synchronized it will stay this way for some time depending on the conditions. In alternating light/dark regimes respecting the cell natural division patterns, the cultures will stay synchronized almost indefinitely. In continuous light, the synchrony will be lost over time, yet, for some time, the divisions will happen at the same time as long as the light conditions are maintained (Figure 8A). Interestingly, although the algae evolved to adapt to the alternating light/dark cycles their cell cycles are not necessarily controlled by circadian rhythms. The link between circadian clock and cell cycle has been established in cyanobacteria [1] and mammalian cells [2,3,4], including cancer cells [4,5]. However, its existence has been a controversial topic in the field of algae dividing by multiple fission. Early reports studying multiple fission cell cycle in *Chlorella* sp. routinely present and use synchronizing experiments with non-circadian duration [6,7]. It has been established that multiple fission cell cycle is regulated by a combination of sizer and timer in *Chlamydomonas reinhardtii* [4,8,9,10,11], but the timer is not circadian. Similar results were also established for another green alga *Desmodesmus quadricauda* [12]. There were reports suggesting involvement of circadian gating of cell division in *Chlamydomonas reinhardtii* [13]. Yet, a comprehensive testing of multiple fission cell cycles at wide range of light intensities and temperatures disregarded the existence of circadian gating in this organism [14,15]. In establishing the synchronization regime, we respected the cell and culture behavior at given growth conditions. The cells were darkened when they started to divide and were re-illuminated when they completed cell division. The synchronization regime we established was in agreement with synchronization regimes used for a relative alga *Chlorella* sp. [6,7].

**Figure 10:**
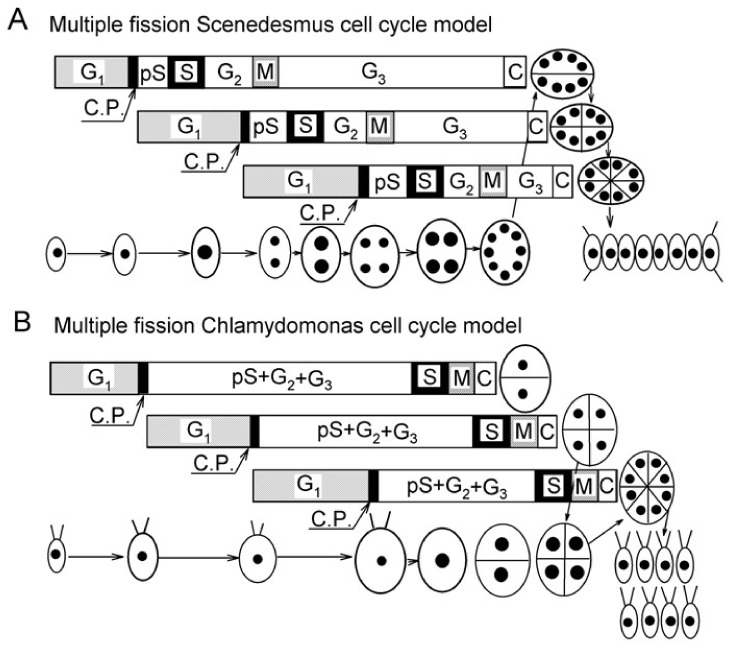

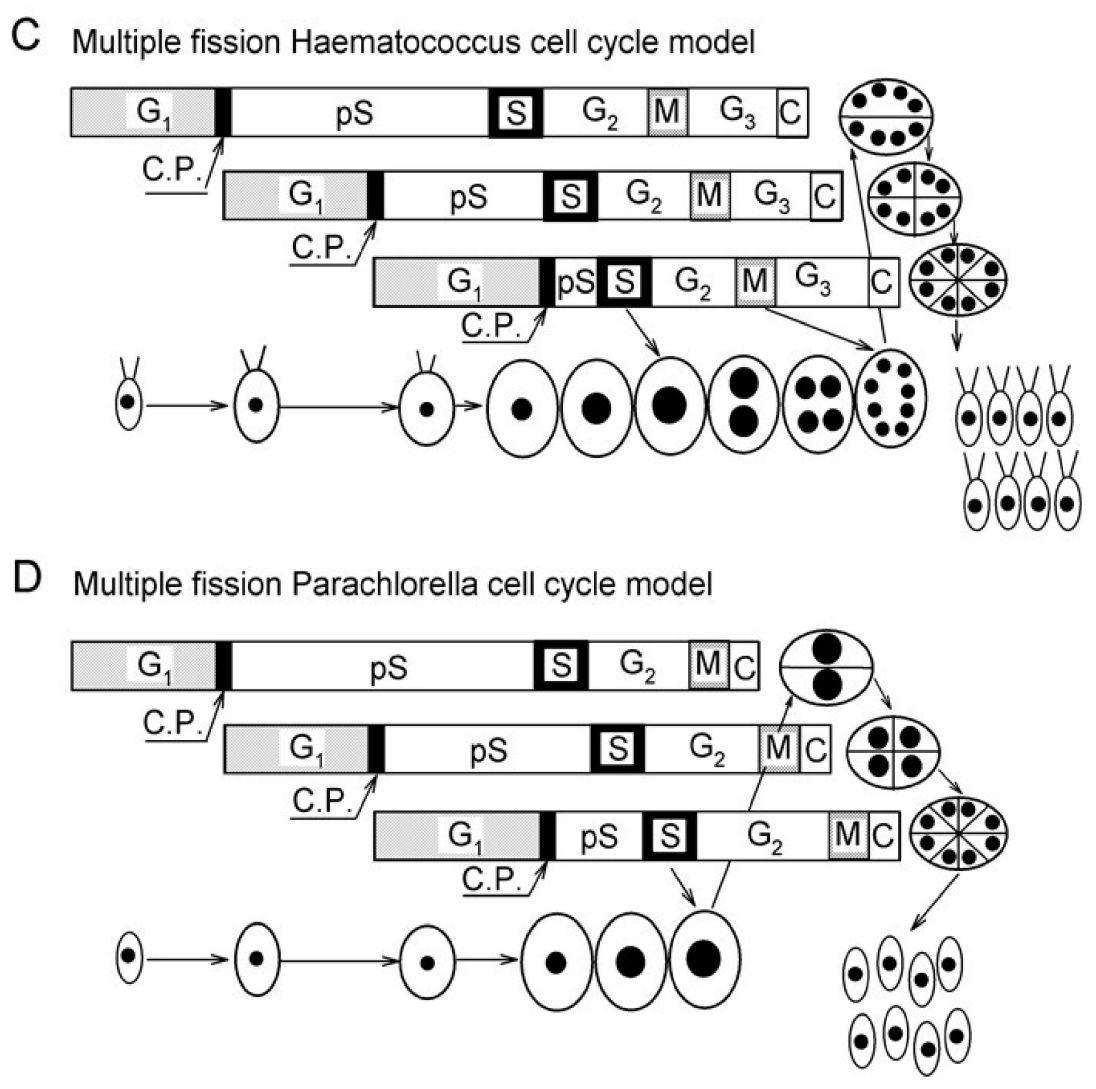
Schematic diagram of growth and the cell cycle in a single cell of different algae dividing into 8 daughter cells. (**A**): *Desmodesmus* (*Scenedesmus*) *quadricauda*, (**B**): *Chlamydomonas reinhardtii*, (**C**): *Haematococcus pluvialis,* (**D**): *Parachlorella kessleri*. The schematic pictures reflect increasing cell size. The full circles inside the cells illustrate the size and number of nuclei. Larger circles indicate a doubling of DNA. Ellipsoids and lines represent protoplast division. Three bars indicate three overlapping growth and reproductive sequences terminated by division into 2, 4, and 8 daughter cells, respectively. Pre-commitment period (G1): the period until threshold critical cell size for commitment to divide (CP) is reached and CP is attained. Post-commitment period consists of pS—the pre-replication phase between CP attainment and the beginning of DNA replication. The processes required for initiation of DNA replication are assumed to happen during this phase. S: DNA replication takes place. G2: the phase between the termination of DNA replication and the start of mitosis (M). Processes leading to the initiation of mitosis are assumed to take place during this phase. G3: the phase separating mitosis from cellular division, which is clearly visible in some algae dividing by multiple fission. The processes leading to cellular division are assumed to take place during this phase. C: the phase during which cell cleavage (protoplast fission) and daughter cell formation occurs. Modified after Zachleder, Schläfli and Boschetti [6], Zachleder and van den Ende [8], Reinecke, Castillo-Flores, Boussiba and Zarka [54] and Zachleder, Bišová and Vítová [3].

Division into more than two daughter cells is common among green algae. Such a multiple fission cell cycle is performed in different species according to different patterns. All patterns share several common features: (1) they are composed of several growth steps, (2) the growth steps run consecutively, (3) completion of each growth step (*n*, *n* ranges from 1 to 10) by doubling the initial biomass will allow for a start of a single reproductive step, (4) each reproductive step or sequence starts with commitment point (CP) and consists of processes of the standard cell cycle; DNA replication, nuclear division, cell division including the gap phases (G1, G2), (5) several (*n*) reproductive steps are running concurrently, (6) cell division is postponed until completion of the final reproductive step and runs simultaneously for all of the reproductive steps leading to a release of 2^*n*^ daughter cells. Two main distinct types of multiple fission cell cycle, consecutive pattern (Scenedesmus—type) and clustered pattern (Chlamydomonas—type) [3,4,6,53] differ in time succession of the reproductive sequences (Figure 10). In each consecutive cell cycle, the attainment of CP is followed shortly by the phase for preparation of DNA replication (pre-S), DNA replication (S), phase of preparation of nuclear division (G2), nuclear division (M), and finally, preparation of protoplast fission (G3) and protoplast fission (C) (Figure 10). This is terminated by formation and release of daughter cells. Such a sequence of events, and the reproductive sequence, repeats after each doubling of cell biomass, i.e., completion of the growth step. Within one multiple fission cell cycle there will be several consecutive doublings of DNA, each followed by a G_2_ phase and then by nuclear division (Figure 10). The overlap among individual reproductive sequences will result in bi-, tetra- and then octa-nuclear cells. Each nuclear division is followed by a G_3_ phase of different duration and thereafter by a corresponding number of protoplast fissions and release of daughter cells [3,4,6,53] (Figure 10A). In the clustered cell cycle pattern, no rounds of DNA replication nor nuclear divisions occur during the majority of the cell cycle. The cells sequentially undergo several growth steps and increase their size correspondingly to the number of growth doublings steps, but they remain haploid and uni-nuclear until the very end of the cell cycle. Then occurs several rounds of DNA replications, each of them immediately followed by corresponding nuclear division. These are followed again nearly immediately by protoplast fission and terminated by the formation of daughter cells. During the entire cell cycle, no polyploid or multi-nuclear stages were present [3,4] (Figure 10B). Besides the two main cell cycle patterns, there could be a combination of these such as the cell cycle pattern of the alga *Haematococcus pluvialis* (class Chlorophyceae, order Volvocales) designated “Haematococcus—type cell cycle” [54]. *H. pluvialis* firstly undergoes DNA replication for all initiated reproductive sequences, which corresponds to the number of daughter cells formed. This way it becomes polyploid. Thereafter it repeatedly divides nuclei becoming poly-nuclear. The poly-nuclear cells divide their protoplasts and form non-flagellated daughter cells inside the mother cell wall. Finally, flagellated daughter cells are released [54] (Figure 10C). The cell cycle of *P. kessleri*, as described in this manuscript, is characterized by multiple rounds of DNA replication (Figure 5). In contrast to the Haematococcus or Scenedesmus pattern, none of the early DNA replications were followed by nuclear division; this only occurred once all replication rounds were terminated. Consequently, the cell became successively polyploid with a single large nucleus (Figure 4). 

From the experiments presented here, the status of the polyploid DNA is not clear. In principle, the replicated copies could stay together in a single chromosome as they do in the polytene chromosome in *Drosophila melanogaster,* or in plants [55,56], or they could undergo endomitosis so that the nuclei contain multiple sets of chromosomes [57]. At the end of the cell cycle, the number of nuclear divisions corresponding to the number of preceding DNA replications are triggered (Figure 3 and Figure 4). 

Each nuclear division is immediately followed by protoplast fission without any intervening G3 phase. In this way no poly-nuclear cells could be observed during the *Parachlorella* cell cycle, similar to the pattern established in *Chlamydomonas* [3,4]. Thus, cells of *P. kessleri* divide by another unique hybrid cell cycle pattern, which is a combination of features of all described patterns of cell cycles in species dividing by multiple fission [3,54] (Figure 10D).

The final biomass content of *P. kessleri* at the end of the cell cycle, measured as the time course of RNA, dry matter and cell volume (Figure 6), increased with light intensity. It multiplied 4-fold within one cell cycle at the lowest irradiance (110 μmol photons m^−2^s^−1^), 12-fold at 250 μmol photons m^−2^s^−1^ and 16-fold at the highest irradiance (500 μmol photons m^−2^s^−1^). Cell growth at any given light intensity did not occur as a continuous increase in the given growth parameter. Rather, it occurred in steps, each of which corresponded to a doubling of the value of the preceding one. The steps were separated by a short interval without (or with only a slight) increase in growth characteristics. With increasing light intensity, growth rate within individual steps increased, the intervening interval without growth shortened and the number of steps increased (Figure 6). These rules were particularly well expressed in the time courses of RNA accumulation (Figure 6A), but they can also be followed for cell volume (Figure 6C) and less so for dry mass (Figure 6B). Such growth with non-overlapping and time-separated steps is typical for multiple fission cell cycle patterns in the majority of chlorococcal and volvocaean algae [3,4,6,8].

Starch accumulates in light via photosynthesis. Under unstressed growth conditions, it serves as a main source of energy and carbon for *P. kessleri* cells kept in the dark or under insufficient light conditions [16,26]. The starch content measured in our experiments represents the net starch content, which is a result of starch synthesis and consumption. Thus clearly, it is an underestimate of the real level of starch synthesis and newly made starch content. The net starch synthesis was negligible in the early part of the cell cycle for the cultures grown at the lowest light intensity (110 μmol photons m^−2^s^−1^) (Figure 7), probably due to the balance between material and energy supply from photosynthesis and their use in synthetic processes [3]. The net starch content as well as its rate of synthesis grew proportionally to the increasing light intensity (Figure 7). The final net content of starch, a 2-fold increase at the lowest light intensity (110 μmol photons m^−2^s^−1^), an 8-fold increase at 250 μmol photons m^−2^s^−1^ and a 10-fold increase at the highest irradiance (500 μmol photons m^−2^s^−1^), were lower than those of RNA (Figure 7, see above). Since nuclear and cellular divisions are the key consumers of starch, even under light conditions [3,27,53,58,59], we assume that part of the newly made starch is immediately spent for reproductive processes. This would also explain why there seems to be significantly less net starch content (only a 10-fold increase) compared to a 16-fold increase in dry mass and cell volume at the highest light intensities. The lower starch levels might be caused by the higher number of reproductive sequences and higher number of daughter cells formed (Figure 5), which naturally requires more energy. The increase in consumption processes also explains the plateau of net starch at the end of the cell cycle in all experimental variants (Figure 7). Moreover, it is known that rates of photosynthesis (and consequently rates of starch synthesis) decrease or are stopped during cell division [60,61]. Neutral lipids represent the other energy reserve of *P. kessleri* [16,26]. In the majority of our experiments, the levels of neutral lipids were negligible (not shown) as they are expected to be synthesized in response to stress conditions [16,26]. This is particularly true for the later phases of stress after starch was (over)produced and is being replaced by neutral lipids as the secondary energy reserve [16,26]. In line with this, we detected more starch in the 70% deuterated culture than in the control but the accumulation of neutral lipid was, for the majority of the experiment, negligible. In contrast, higher levels of neutral lipids were found in the cultures grown in 99% D_2_O (Figure 8D and Figure 9). Moreover, the increase in the amount of neutral lipids increased with light intensity and was most strongly manifested in the cultures pre-adapted to 99% D_2_O (Figure 8D). The same cultures also accumulated the most starch (Figure 9), suggesting they are stressed but still able to cope with the stress levels [16,26].

Cultivation in increasing concentrations of deuterated water showed a distinct effect on cell division and cell growth, with growth being less affected than cell division (Figure 8, compare A and B, compare Table 1 and Table 2). In the presence of deuterated water, the response to different light intensities was opposite to its effect in normal water, i.e., the increasing light intensity decreased growth rates (Figure 8). This was probably due to the combined effect of light and deuterium stress on the cells and is particularly clearly seen for the non-adapted 99% culture (Figure 8C). There was a significant degree of variability of Fv/Fm ratio, which suggested some of the cultures acclimated to the presence of deuterium. The adaptation process was faster in 70% cultures where it took approximately 48 h to recover the Fv/Fm ratios in the lower light intensities but 96 h at the highest light intensity. In 99% cultures, the cultures at the two lower light intensities adapted by 96 h of the treatment, whilst the ones at the higher light intensities (300 and 400 μmol photons m^−2^s^−1^) were unable to recover and eventually died (Figure 8C). Clearly, the cells were able to adapt even to the highest concentrations of deuterium as it is evident from the cultures adapted at 99%. Such cultures did not show a decline in Fv/Fm ratios at lower light intensities. At higher light intensities (300 and 400 μmol photons m^−2^s^−1^), there was a drop in Fv/Fm ratios comparable to that in 70% cultures at the highest light intensities (Figure 8C). Yet, such cultures were able to recover the initial Fv/Fm values within two days (Figure 8C). Interestingly, there was only a small effect of light intensity on the number of daughter cells in different concentrations of deuterated water, further supporting the smaller effect of deuterium on growth. Although mass multiplication was less affected than division number in all variants, the mass doubling time was similar for all light intensities (Table 2) and it approximately correlated with cell number doubling time of the same culture (compare Table 1 and Table 2). The cells show striking resilience to the presence of deuterated water. In the presence of 70% of deuterated water they recovered from the stress within 48 h (Figure 8C). They were even able to cope quite well with 99% deuterated water provided they are pre-adapted to this concentration (Figure 8C). How growth in 99% deuterated water for a prolonged period of time affects cell physiology is a matter for future research. Particularly interesting will be the mechanisms allowing cells to cope with the majority of their hydrogen being replaced by deuterium as is the case of pre-adapted 99% cultures. Due to the inhibitively high price of deuterated water, the treatment with deuterated water is quite expensive. It can be justified in the field of basic research to understand the effects of deuterium on the cell metabolism. Understanding the stress reaction is important even though high concentrations of deuterated water are never present in nature. As stable isotopic labeling is extensively used to analyze cell metabolism [31,32,62] it is crucial to disentangle the effects of the deuterium tracer in the cells to discriminate its effect from the effect of the treatment. Furthermore, although treatment with deuterared water would be too expensive to be used in large scale algal biomass production it is fully justified for production of very high added value products such as deuterated compounds [31,39,40]. The individual isolated biomolecules can be used as analytical standards or as a well-defined source of deuterium for stable isotopic labeling. Indeed, algae were shown to serve as a commercial source of fully deuterated sugars and amino acids [63], proteins [64], chlorophylls [39,65], and carotenoids [39]. The fully deuterated algal biomass arising from the experiments presented here can be used for production of deuterated starch and, to a certain extent, neutral lipids but also other cellular components.

## 5. Conclusions

We describe a novel pattern of multiple fission cell cycle. The pattern utilized by cells of *P. kessleri* consists of multiple rounds of DNA replication, which, after completion of the last one, are followed by successive nuclear divisions. Each of the nuclear divisions was immediately followed by cell division. Thus, morphologically, the cells became sequentially polyploid but not polynuclear. In this way, the cell cycle pattern is yet another combination of established patterns of multiple fession cell cycles. Cultivation under increasing light intensities led to an increase both in growth rate and the number of growth steps completed by the cells, leading to a higher number of daughter cells being formed. Growth of *P. kessleri* in different concentrations of heavy water slowed growth and progression of the cell cycle, with cell division being more affected than cell growth. The inhibitory effect of heavy water was more pronounced with increasing light intensity. The highest concentration of heavy water (99%) caused a prolonged stress that led to the accumulation of neutral lipids. However, the stress levels can be decreased by pre-adaptation of the cells to high concentrations of heavy water. Our results establish *P. kessleri* as a novel model of the cell cycle for basic research as well as to set a baseline for extension of its biotechnological potential for the production of deuterated compounds.

## Figures and Tables

**Table 1:** Comparison of division number and cell number doubling times for cultures grown in medium containing different concentrations of deuterated water at different light intensities. The average of at least three experiments is shown. Asterisks (*) denote cultures pre-adapted for one week to a given concentration of deuterated water, NA, the calculations are not reliable due to cell death and lysis.

Concentration of Deuterated Water (%)	Light Intensity (μmol Photons m^−2^s^−1^)	Division Number	% of Control	Doubling Time	Fold Retardation of Control
0	150	6.96	100	9.98	1
	200	7.44	100	9.05	1
	300	8.27	100	8.79	1
	400	9.35	100	7.98	1
70	150	4.77	68.53	22.80	2.28
	200	4.54	61.00	13.35	1.48
	300	3.92	47.45	11.17	1.27
	400	4.76	50.85	9.28	1.16
99	150	1.33	19.07	117.32	11.75
	200	1.38	18.49	86.27	9.53
	300	1.64	19.88	158.68	18.04
	400	1.14	12.23	145.78	18.28
99 *	150	1.36	19.52	34.32	3.44
	200	1.12	15.05	46.13	5.10
	300	1.20	14.48	NA	NA
	400	1.43	15.31	NA	NA

**Table 2:** Comparison of mass multiplication factor and mass doubling times for cultures grown in medium containing different concentrations of deuterated water at different light intensities. Average of at least three experiments is shown. Asterisk (*) denotes cultures pre-adapted for one week to given deuterated water concentration.

Concentration of Deuterated Water (%)	Light Intensity (μmol Photons m^−2^s^−1^)	Mass Multiplication Factor	% of Control	Mass Doubling Time (h)	Fold Retardation of Control
0	150	4.33	100	12.79	1
	200	5.65	100	11.65	1
	300	5.54	100	13.65	1
	400	5.74	100	10.60	1
70	150	3.64	84.13	15.29	1.20
	200	3.42	60.52	13.75	1.18
	300	3.32	59.86	16.04	1.18
	400	4.03	70.27	13.24	1.25
99	150	1.83	42.39	118.23	9.24
	200	1.29	22.73	142.92	12.26
	300	1.13	20.43	306.28	22.44
	400	1.05	18.23	309.10	29.17
99 *	150	3.18	73.46	42.53	3.33
	200	2.78	49.20	47.62	4.09
	300	2.78	50.25	49.99	3.66
	400	2.11	36.68	49.09	4.63

## Data Availability

Not applicable.
